# P-1628. Cause Of COVID-19 Outbreaks in Outdoor Sports: A Systematic Review

**DOI:** 10.1093/ofid/ofaf695.1804

**Published:** 2026-01-11

**Authors:** Masaki Machida, Koichi Dai, Itaru Nakamura, Shigeru Inoue

**Affiliations:** Tokyo Medical University, Shinjuku-ku, Tokyo, Japan; Tokyo Medical University Hospital, Shinjuku-ku, Tokyo, Japan; Tokyo Medical University Hospital, Shinjuku-ku, Tokyo, Japan; Tokyo Medical University, Shinjuku-ku, Tokyo, Japan

## Abstract

**Background:**

In sports activities, respiratory viral infections, such as coronavirus disease 2019 (COVID-19) are more likely to outbreak owing to group activities, opportunities for contact with individuals, and vocalisations. During the COVID-19 pandemic, several cases of COVID-19 outbreaks during various sports and exercise were reported. However, the common causes of these outbreaks remain unclear. Outdoor sports, with better ventilation, generally present a lower risk of outbreaks than indoor sports; however, outbreaks associated with outdoor sports have been reported. In this review, we aimed to systematically identify COVID-19 outbreaks during outdoor sports and clarify their causes.

**Figure:**
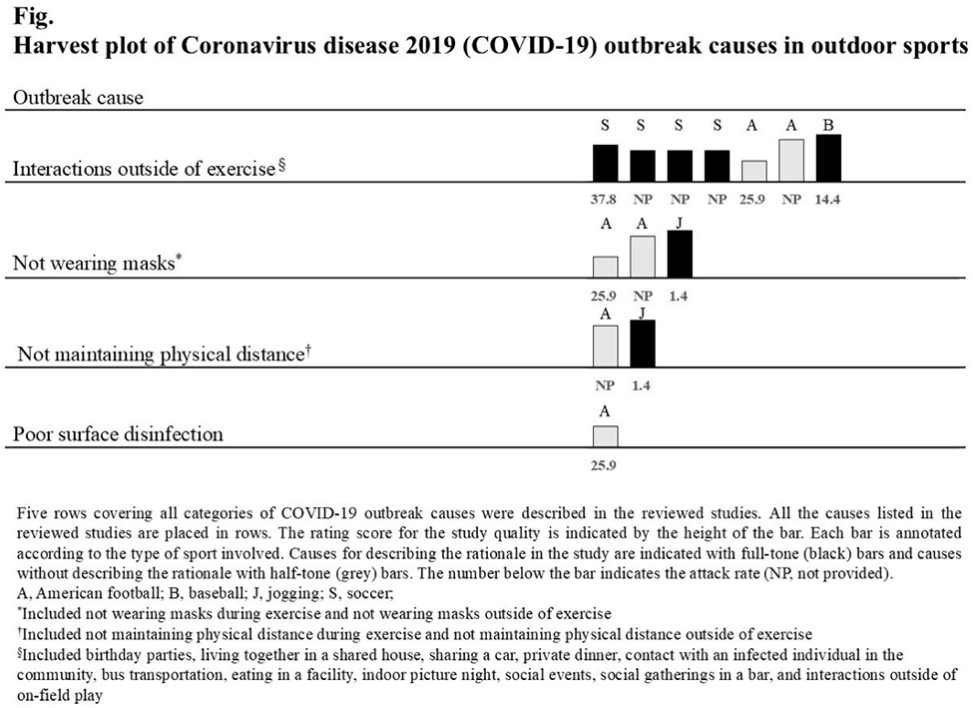

**Methods:**

The eligibility criteria were published articles reporting case investigations on COVID-19 outbreaks during outdoor sports and their causes. Studies such as reviews and observational studies without case investigations were excluded. PubMed, CINAHL, WHO COVID-19 Research Database, and Ichushi Web were searched on August 28, 2023. The quality of the included works was rated using a quality criteria checklist adapted from a previous systematic review of influenza outbreaks. Vote counting for outbreak causes was performed according to the sport type. This study was registered in PROSPERO.

**Results:**

Seven articles reporting eight outbreaks were identified. The number of reported outbreaks was highest in the soccer, followed by American football, baseball and jogging. In many cases, the participants did not wear masks during sports; furthermore, they performed physical condition self-checks or underwent periodic laboratory screening. The cause of all outbreaks, except for those involving jogging, was interaction outside the exercise, such as social events (i.e., parties and car sharing). The outbreaks during jogging were reported to be caused by not wearing masks and not maintaining physical distance; however, the attack rate was very low (1.4%).

**Conclusion:**

We found a limited number of case investigations suggesting that COVID-19 outbreaks during outdoor sports may be associated with interactions outside of the game. Prevention strategies focused on mitigating this issue may be effective in preventing respiratory infectious diseases outbreaks in outdoor sports.

**Disclosures:**

All Authors: No reported disclosures

